# Sustainable Valorization of Farmed Atlantic Salmon Processing By-Products: Supercritical CO_2_ Extraction of Lipid Fraction and pH-Shift Recovery of Proteins from the Residual Material

**DOI:** 10.3390/foods15213947

**Published:** 2026-11-03

**Authors:** Isidora Vlaović, Milica Perović, Branislava Đermanović, Slađana Krivošija, Vanja Travičić, Jelena Vulić, Senka Vidović

**Affiliations:** 1 Faculty of Technology Novi Sad, University of Novi Sad, Boulevard Cara Lazara 1, 21000 Novi Sad, Serbia; isidora.vlaovic@uns.ac.rs (I.V.); perovicmilica@uns.ac.rs (M.P.); branislava.djermanovic@fins.uns.ac.rs (B.Đ.); sladjana.krivosija@uns.ac.rs (S.K.); jvulic@uns.ac.rs (J.V.); senka.vidovic@uns.ac.rs (S.V.); 2 Institute of Food Technology in Novi Sad, University of Novi Sad, Bulevar Cara Lazara 1, 21000 Novi Sad, Serbia

**Keywords:** Atlantic salmon, skin-on trimmings, supercritical CO_2_ extraction, ultrasound-assisted pH-shift, astaxanthin, protein isolate, circular bioeconomy

## Abstract

Farmed Atlantic salmon skin-on trimmings were valorized through a sequential process combining supercritical carbon dioxide (SC-CO_2_) extraction with protein recovery from the resulting defatted residue. Freeze-dried material was extracted at 40 °C and 100, 200, and 300 bar for 540 min to evaluate the effect of pressure on extraction yield. The highest yield among the tested conditions was obtained at 300 bar (28.08%), and this extract was selected for further characterization. It contained 98.55 g/100 g lipids, with oleic acid as the predominant fatty acid, together with EPA, DHA, and astaxanthin (215.43 µg/mL), and showed concentration-dependent ABTS radical-scavenging activity (IC_50_ ≈ 109 mg/mL). The corresponding defatted biomass was subsequently subjected to conventional and ultrasound-assisted pH-shift protein recovery. Ultrasound assistance increased protein yield from 36.70 to 47.91 g/100 g. Both isolates contained more than 91 g/100 g protein and had similar amino acid profiles, with essential amino acids accounting for approximately 41% of total amino acids. Ultrasound treatment also improved protein solubility, water-holding capacity, and ABTS radical-scavenging activity, whereas the conventionally recovered isolate showed higher oil-holding capacity. These results support the sequential recovery of lipid- and protein-rich fractions from the same salmon-processing by-product.

## 1. Introduction

Global food demand is projected to increase substantially by 2050 as a consequence of population growth, urbanization, and changing dietary patterns, placing increasing pressure on food production systems and natural resources [1]. At the same time, large quantities of food-processing by-products are generated annually, many of which remain insufficiently utilized despite containing valuable nutrients and bioactive compounds. Recovering these constituents and converting them into high-value products has therefore become an important component of circular bioeconomy strategies aimed at improving resource efficiency and reducing environmental burdens [2,3].

The seafood industry is among the largest producers of nutrient-rich by-products. Depending on the species and processing method, heads, frames, skin, viscera, roe, and trimmings may represent up to 60% of the processed biomass [4]. During Atlantic salmon filleting, approximately one-quarter of the original fish mass is removed as processing by-products, including skin-on trimmings generated during fillet preparation [5]. These trimmings consist of skin with attached muscle tissue and contain considerable amounts of both lipids and proteins [6,7]. Nevertheless, they are commonly utilized for relatively low-value purposes, although their composition makes them suitable for the recovery of valuable ingredients [4,8].

The lipid fraction of Atlantic salmon is characterized by a high content of unsaturated fatty acids, particularly the long-chain omega-3 polyunsaturated fatty acids eicosapentaenoic acid (EPA) and docosahexaenoic acid (DHA), together with astaxanthin, the carotenoid responsible for the characteristic red coloration of salmon flesh [9,10]. These compounds are of considerable interest because of their nutritional and technological value, but their recovery requires extraction techniques that minimize oxidative degradation and thermal damage [11,12]. Supercritical carbon dioxide (SC-CO_2_) extraction has become one of the most attractive alternatives for recovering lipophilic compounds from marine biomass owing to its moderate operating temperatures, oxygen-free environment, and solvent-free extracts [13]. Furthermore, extraction pressure strongly influences CO_2_ density and solvent power, thereby affecting both extraction efficiency and the composition of the recovered lipid fraction [14,15].

Following lipid extraction, the remaining biomass still represents a valuable source of proteins. Fish proteins recovered from salmon-processing by-products possess a balanced amino acid composition and exhibit functional properties such as high solubility, water-holding capacity, emulsifying ability, and gel-forming potential, making them attractive ingredients for food applications [16,17]. Among the available recovery techniques, pH-shift processing has proven particularly effective because it enables selective protein solubilization followed by isoelectric precipitation. More recently, ultrasound has been incorporated into the pH-shift process to enhance protein extraction and modify the physicochemical and functional properties of the resulting protein isolates [18,19,20,21].

Previous studies have investigated the recovery of lipid fractions from salmon-processing by-products using SC-CO_2_ extraction and, separately, the recovery of proteins by pH-shift processing, including ultrasound-assisted approaches [8,16,17,18,19,20,21]. However, these valorization routes have predominantly been studied independently. Consequently, the direct use of defatted biomass generated after SC-CO_2_ extraction as a feedstock for subsequent pH-shift protein recovery, together with the characterization of both lipid- and protein-rich fractions obtained from the same raw material, remains insufficiently explored. Therefore, the novelty of the present study lies in the direct use of SC-CO_2_-defatted biomass as the feedstock for subsequent conventional and ultrasound-assisted pH-shift protein recovery within a single integrated valorization strategy.

## 2. Materials and Methods

### 2.1. Raw Material and Chemicals

Atlantic salmon (*Salmo salar*) skin-on trimmings generated during filleting were obtained from Vasović d.o.o. (Čačak, Serbia), a company for fish and seafood products. The raw material originated from farmed Atlantic salmon of Norwegian origin and was supplied as vacuum-packed, frozen pieces. Samples were stored at −18 °C and subsequently freeze-dried using an Alpha 2-4 LSC freeze dryer (Martin Christ, Osterode am Harz, Germany). The freeze-dried material was ground using an analytical batch mill (IKA A11 basic S000, IKA-Werke GmbH & Co. KG, Staufen, Germany) before supercritical CO_2_ extraction and comparative fatty acid analysis.

Commercial CO_2_ (purity 99.9%, *v*/*v*) supplied by Messer (Novi Sad, Serbia) was used for supercritical extraction. All other chemicals used during the experiment were analytical-grade reagents.

### 2.2. Supercritical CO_2_ Extraction

The high-pressure extraction was performed using a laboratory high-pressure extraction system (HPEP, NOVA-Swiss, Effretikon, Switzerland) with SC-CO_2_ as the extraction solvent. Three independent extraction runs were performed at each investigated pressure (100, 200, and 300 bar), using 50 g of freeze-dried and ground salmon skin-on trimmings per extraction run at 40 °C. The investigated pressure range was selected to evaluate the effect of increasing CO_2_ density and solvent strength on lipid recovery and was consistent with the pressure range previously investigated for SC-CO_2_ extraction of Atlantic salmon-processing by-products [13,14].

Extraction yield (EY) was measured at 0.5, 1, 1.5, 2, 3, 4, 5, 6, 7, 8, and 9 h to allow the extraction kinetics to be monitored over an extended period and to determine the stage at which the increase in yield became progressively smaller. During extraction, the pressure and temperature in the separator unit were 15 bar and 23 °C, while the flow rate was set at 1.94 kg/h. Using this procedure, three independent SC-CO_2_ extraction runs were conducted at each investigated pressure (100, 200, and 300 bar). The extracts were protected from light and stored at −18 °C until analysis. The extraction yield (EY) at each sampling time was calculated as the cumulative mass of the collected extract relative to the initial mass of freeze-dried raw material loaded into the extractor according to Equation (1):

EY (%) = (m_e_/m_0_) × 100 (1)

where m_e_ is the cumulative mass of extract collected at the corresponding sampling time (g), and m_0_ is the initial mass of freeze-dried raw material loaded into the extractor (50 g).

#### Chemical Composition of the Raw Material and SC-CO_2_ Extract

The chemical composition of the freeze-dried salmon skin-on trimmings and the SC-CO_2_ extract obtained at 300 bar was determined using standard ISO methods. Moisture content was determined according to ISO 1442:1997 [22], while protein content was determined by the Kjeldahl method following ISO 937:1978 [23], using a nitrogen-to-protein conversion factor of 6.25. Fat content was determined according to ISO 1443:1973 [24]. Ash content of the freeze-dried raw material and the SC-CO_2_ extract was determined according to ISO 936:1998 [25]. For the SC-CO_2_ extract, ash content was below the detection limit of the applied gravimetric method (LOD = 0.043 g/L; LOQ = 0.1 g/L) and was therefore reported as not detected (ND). Carbohydrate content was calculated by difference [26]. Analyses were performed in triplicate, and the results were expressed as g/100 g of sample.

### 2.3. Lipid Extraction and Fatty Acid Composition Analysis by GC-FID

Lipids were extracted from the samples following the cold extraction procedure [27], employing a chloroform–methanol mixture (2:1, *v*/*v*) as the extraction solvent. After 2.5 h of extraction, the solvent phase containing the lipids was filtered and concentrated under vacuum in a Binder VD 115 dryer (Binder, Tuttlingen, Germany) until complete solvent removal. The recovered oil was immediately flushed with nitrogen to reduce oxidative changes before further analysis.

Fatty acid methyl esters (FAMEs) were then prepared in accordance with the ISO 12966-2:2017 standard [28]. Fatty acid determination was performed by gas chromatography using an Agilent 7890A instrument (Agilent Technologies, Santa Clara, CA, USA) coupled with a flame ionization detector (FID). Separation of FAMEs was achieved on an SP-2560 fused silica capillary column (100 m × 0.25 mm i.d., 0.20 μm film thickness; Supelco, Bellefonte, PA, USA), while helium served as the carrier gas. One microliter of each derivatized sample was introduced into the chromatographic system using a split injection mode (1:30). The injector and detector temperatures were both set at 250 °C. The oven temperature program began at 140 °C with a 3 min isothermal period, followed by a temperature ramp of 3 °C/min to 220 °C and a 5 min hold. Subsequently, the temperature was increased to 240 °C at 2 °C/min and maintained for 10 min. Fatty acids were assigned by matching the retention times of the chromatographic peaks with those obtained from the Supelco 37 Component FAME Mix reference standard (Sigma-Aldrich, St. Louis, MO, USA). Fatty acid composition was expressed as relative percentages of total fatty acids using peak-area normalization. A chromatographic peak that could not be matched with the available reference standard was reported as an unidentified fatty acid and was excluded from the calculation of fatty acid-group sums and nutritional indices.

### 2.4. Astaxanthin Content—HPLC

Quantification of astaxanthin content in the supercritical CO_2_ extract obtained at 300 bar was performed by HPLC on Shimadzu Prominence equipment (Shimadzu, Kyoto, Japan), which contains an LC-20AT binary pump, CTO-20A thermostat, and SIL-20A automatic dispenser connected to a DAD detector. The separation was performed on a GRACE-Vydac 201TP54 C18 column, 250 × 4.6 mm, 5 mm (Hesperia, CA, USA). Briefly, 250 µL of the extract was diluted with 250 µL tetrahydrofuran (THF) and 250 µL methanol to ensure complete solubilization of carotenoids. The resulting solution was filtered through a 0.22 µm PTFE syringe filter, and 50 µL was injected into the chromatographic system. The solvent used as the mobile phase was analytical-grade methanol: stabilized THF (95:5, *v*/*v*) containing 0.01% BHT; the flow rate was 1 mL/min. Astaxanthin was detected at 475 nm. The wavelength range used was 200–600 nm. Quantification was performed using an external calibration curve prepared with astaxanthin standards.

### 2.5. ABTS Assay

The ability of the supercritical CO_2_ extract to neutralize ABTS•^+^ radicals was evaluated using the spectrophotometric method [29], with slight modifications [30]. A stock solution of ABTS was prepared by combining a 7 mM aqueous solution of 2,2′-azino-bis(3-ethylbenzothiazoline-6-sulfonic acid) diammonium salt (ABTS) with 2.45 mM potassium persulfate in a 1:1 (*v*/*v*) ratio, then incubating for 16 h at room temperature in the dark. This stock solution was diluted with ethanol to obtain an absorbance of 0.70 ± 0.02 at 734 nm. The extract was dissolved in ethyl acetate and tested at five concentrations (40, 60, 80, 100, and 120 mg/mL). A volume of 100 µL of each extract solution was mixed with 2900 µL of the ABTS working solution. The mixtures were incubated for 300 min at room temperature in the dark, after which the absorbance was measured at 734 nm. The extended incubation period was selected based on previously reported modifications of the ABTS assay for lipophilic extracts, allowing sufficient interaction between the ABTS radical cation and antioxidant constituents of the lipid-rich sample. All reaction mixtures were maintained in the dark at room temperature to minimize potential oxidative changes during incubation. The control was prepared using 100 µL of ethyl acetate instead of the extract solution. Measurements were performed in triplicate. ABTS radical-scavenging activity was calculated according to Equation (2):

ABTS radical-scavenging activity (%) = [(A_0_ − A_1_)/A_0_] × 100 (2)

where A_0_ is the absorbance of the control, and A_1_ is the absorbance of the sample. The IC_50_ value was estimated by linear regression of the concentration–response curve.

### 2.6. Extraction of Proteins

#### 2.6.1. pH-Shift Extraction

Protein was isolated from salmon by-products using the pH-shift extraction method according to the procedure [17]. Dried salmon residues, remaining after supercritical extraction, were homogenized with cold distilled water, and the pH of the homogenate was adjusted to 11.0 using 2 M NaOH to promote alkaline protein solubilization. The pH-adjusted mixture was shaken on ice for 30 min and subsequently centrifuged at 8000 rpm (Sorvall RC-5B) for 15 min to separate the soluble protein. The supernatant was collected and subjected to isoelectric precipitation by adjusting the pH to 5.5 with 1 M HCl. Following precipitation, the sample was centrifuged again at 8000 rpm for 15 min to recover the protein precipitate. The obtained protein was freeze-dried at −45 °C for 48 h (Martin Christ Alpha LSC 2–4, Osterode, Germany), and the resulting protein isolate was subsequently characterized.

#### 2.6.2. Ultrasound-Assisted pH-Shift Extraction

The material was prepared as described above, suspended in distilled water, and the pH was adjusted to 11.0 using 2 M NaOH. Ultrasound-assisted extraction was performed using an ultrasonic homogenizer (JY96-IIN, Ningbo Scientz Biotechnology Co., Ltd., Ningbo, China) equipped with a titanium cylindrical probe (6 mm diameter). During sonication, the sample vessel was placed in an ice bath to minimize heat generation and prevent thermal denaturation of the dissolved proteins. The probe was submerged to 1/3rd of the suspension depth. Ultrasound was applied at a frequency of 25 kHz using a power of 250W, with a pulsed mode consisting of 5 s of ultrasonic irradiation followed by a 5 s clearance interval (50% duty cycle), for a total treatment time of 20 min. The temperature protection limit was set at 24 °C. After the ultrasound treatment, the pH-shift extraction procedure was conducted under the same conditions, and the remaining steps were performed according to the protocol described above.

### 2.7. Determination of Chemical Composition

The chemical characterization of the proteins included the determination of moisture, protein, and ash content. Protein content was determined using the Kjeldahl method [AOAC 991.20]. Ash content was determined by a gravimetric method, through drying at 550 °C until a constant mass was achieved. Moisture content was determined by oven drying at 85 °C to constant weight.

### 2.8. Characterization of Extracted Proteins

#### 2.8.1. Determination of Protein Solubility

The protein sample was dissolved in distilled water at pH 9 to a final concentration (C1) of 10 mg/mL and thoroughly mixed on a shaker at room temperature for 4 h, after which the pH was adjusted to 9 if necessary. The suspension was left overnight at 4 °C for better protein hydration, and insoluble particles were separated by centrifugation at 10,000 rpm for 10 min. The concentration of dissolved proteins in the supernatant (C2), measured by the Bradford method [31], was used to calculate solubility according to Equation (3):

Solubility (%) = C2/C1×100(3)

#### 2.8.2. Water and Oil Holding Capacity

Water holding capacity (WHC) and oil holding capacity (OHC) of protein isolates were determined according to Tan, Ying-Yuan, and Gan [32]. The WHC and OHC were expressed as grams of water or oil held per gram of protein isolate.

#### 2.8.3. Determination of Antiradical Scavenging Activity

The antioxidant activity of salmon protein isolates was determined as 2,2′-azinobis-(3-ethylbenzothiazoline-6-sulfonic acid) (ABTS•+) radical scavenging activity according to the method [29] with slight modifications. Protein isolates were dissolved in phosphate-buffered saline (PBS), pH 7.0, to obtain a final concentration of 2 mg/mL. A 50 μL sample was mixed with 950 μL of the diluted ABTS radical solution, mixed on a vortex for 30 s and incubated in a dark environment for 6 min at room temperature. The control solution was prepared using 50 μL of PBS instead of the sample, while PBS was used as the blank. Absorbance was measured at 734 nm, and ABTS radical scavenging activity (%) was calculated using Equation (2).

#### 2.8.4. Determination of Amino Acid Composition

Amino acid composition analysis of the obtained proteins was performed using ion exchange chromatography with an Automatic Amino Acid Analyzer Biochrom 30 + (Biochrom, Cambridge, UK), following the method of Tomičić et al. [33]. The technique involved amino acid separation using strong cation exchange chromatography, followed by the ninhydrin color reaction and photometric detection at 570 nm, except for proline, which was detected at 440 nm. Samples were previously hydrolyzed in 6 M HCl (Merck, Germany) and, for tryptophan determination, in 6 M NaOH at 110 °C for 24 h. After hydrolysis, the samples were cooled to room temperature and dissolved in 25 mL of loading buffer (pH 2.2) (Biochrom, Cambridge, UK). The prepared samples were then filtered through a 0.22 μm pore-size PTFE filter (Plano, TX, USA), and the filtrate was transferred into a vial (Agilent Technologies, USA) and stored in a refrigerator prior to analysis. The amino acid peaks were identified by comparing their retention times with those of corresponding amino acid standards purchased from Sigma-Aldrich (Amino Acid Standard, Sigma-Aldrich, St. Louis, MO, USA). The results were expressed as the mass of amino acid (g) per 100 g of protein.

#### 2.8.5. Fourier-Transform Infrared Spectroscopy

Fourier-transform infrared (FTIR) spectra of the freeze-dried protein isolates obtained by conventional and ultrasound-assisted pH-shift processing were recorded using a Shimadzu IRaffinity-1S Fourier-transform infrared spectrometer (Shimadzu, Kyoto, Japan) equipped with a MIRacle 10 attenuated total reflectance accessory (ATR-FTIR; Dia/ZnSe). Protein powder was flattened against the ATR crystal to ensure adequate contact with the crystal surface. Spectra were collected over the wavenumber range of 4000–400 cm^−1^ at a spectral resolution of 4 cm^−1^ by averaging 40 scans for each sample. The spectra were used to identify the characteristic absorption bands of the recovered proteins and to evaluate possible changes in protein conformation associated with ultrasound treatment.

### 2.9. Statistical Analysis

Data are presented as mean ± standard deviation, with the type and number of replicates specified where appropriate. Extraction yield (EY) results were obtained from three independent SC-CO_2_ extraction runs and are presented as mean ± standard deviation (SD). Where appropriate, statistical differences were evaluated by one-way ANOVA followed by Tukey’s test (*p* < 0.05) using Statistica (TIBCO Software Inc., Palo Alto, CA, USA).

## 3. Results and Discussion

### 3.1. Extraction Yield

SC-CO_2_ extraction was used as the first step in the sequential valorization of Atlantic salmon skin-on trimmings to recover the lipid-rich fraction. Due to the selectivity of SC-CO_2_ toward low-polarity compounds, lipids and associated lipophilic constituents were extracted, whereas the protein fraction remained in the defatted biomass and was subsequently recovered by pH-shift processing, with and without ultrasound assistance. Pressure strongly influences CO_2_ density and solvent strength and can therefore affect extraction yield and extract composition [34]. For this reason, extractions were performed at 100, 200, and 300 bar, while the temperature and CO_2_ flow rate were kept constant. The cumulative extraction yields obtained as a function of extraction time are presented in Table 1.

The cumulative extraction yield increased with extraction time at all investigated pressures and was consistently highest at 300 bar. After 540 min, yields of 3.47%, 9.65%, and 28.08% were obtained at 100, 200, and 300 bar, respectively, showing a clear pressure dependence. A similar trend was reported by Adeoti and Hawboldt [34] during SC-CO_2_ extraction of freeze-dried Atlantic salmon processing waste composed of heads, trimmings, and frames. In their study, the extraction yield increased over the investigated pressure range of 150–350 bar, with the highest yield obtained at 350 bar. Although the raw material and operating conditions differed from those used in the present study, both studies showed improved lipid recovery at higher extraction pressures.

At 300 bar, the increase in extraction yield became progressively smaller toward the end of the process. The cumulative yield increased from 25.99% at 420 min to 28.08% at 540 min, while only 0.46 percentage points were recovered during the final 60 min. This suggests that most of the readily extractable lipid fraction had already been recovered by the later stages of extraction. From the perspective of industrial throughput, 420 min may therefore represent a more practical extraction endpoint, as approximately 92.6% of the final yield obtained after 540 min was already recovered at this time, while further extraction resulted in only a limited increase in yield. Since 300 bar provided the highest final yield, the extract and the corresponding defatted biomass obtained under this condition were selected for further characterization and protein recovery.

### 3.2. Proximate Composition of the Salmon Trimmings and SC-CO_2_ Extract

To assess the composition of the starting material and the degree of lipid enrichment achieved by SC-CO_2_ extraction, the composition of the freeze-dried salmon skin-on trimmings and the extract obtained at 300 bar was determined (Table 2). The freeze-dried raw material contained 54.42 g/100 g protein and 40.14 g/100 g lipids, showing that this processing side stream represents a rich source of both major fractions. Following SC-CO_2_ extraction, the recovered extract consisted predominantly of lipids, which accounted for 98.55 g/100 g, while its moisture and protein contents were only 0.76 and 0.46 g/100 g, respectively. These results show that SC-CO_2_ extraction produced a highly concentrated lipid fraction, while the high protein content of the starting material supported the subsequent recovery of proteins from the defatted biomass.

The composition observed in the present study is consistent with the considerable variation reported among Atlantic salmon processing co-products, whose protein and lipid contents depend strongly on the anatomical fraction and sample preparation. Malcorps et al. [7] reported a lipid content of 26.4 g/100 g on a wet-weight basis for Atlantic salmon trimmings, while Ulleberg et al. [35] reported approximately 10% fat and 22% protein in deboned meat and 34% fat and 15% protein in belly flaps. Direct numerical comparison with the present results is limited because the samples differed in anatomical composition and because the skin-on trimmings used in the present study were freeze-dried and contained only 1.07% residual moisture. Nevertheless, the high protein and lipid contents show that freeze-dried skin-on trimmings represent a concentrated source of both fractions and support their use as a raw material for sequential lipid and protein recovery.

### 3.3. Fatty Acid Composition

Fatty acids are among the most important components of fish oil due to their nutritional value and significant impact on human health. In recent decades, particular attention has been devoted to the quality of dietary fatty acids, with emphasis on unsaturated fatty acids, especially monounsaturated fatty acids (MUFAs) and polyunsaturated fatty acids (PUFAs). Numerous studies have demonstrated that a higher intake of unsaturated fatty acids contributes to the maintenance of cardiovascular health, regulation of lipid metabolism, and reduction of the risk of chronic non-communicable diseases, whereas excessive consumption of saturated fatty acids has been associated with elevated LDL cholesterol levels and an increased risk of atherosclerosis and other cardiovascular disorders [9,36]. Long-chain omega-3 fatty acids, particularly eicosapentaenoic acid (EPA) and docosahexaenoic acid (DHA), exhibit anti-inflammatory, antithrombotic, and cardioprotective properties and play essential roles in the development and functioning of the nervous system [9]. Salmon is recognized as one of the most important dietary sources of omega-3 fatty acids; however, its fatty acid composition is strongly influenced by fish origin and feed composition. Over recent decades, substantial changes in the lipid profile of farmed salmon have occurred due to the progressive replacement of fish oil with vegetable oils in aquaculture feeds. Consequently, farmed salmon generally contains higher proportions of oleic (C18:1n9), linoleic (C18:2n6), and α-linolenic acid (C18:3n3), while the relative proportions of EPA and DHA are typically lower than those observed in wild populations [37,38].

The results obtained in the present study are consistent with the fatty acid profile characteristic of modern farmed Atlantic salmon and are shown in Table 3.

In both lipid fractions, MUFAs were the predominant group, accounting for 49.41–50.91% of total fatty acids, with oleic acid representing the most abundant individual fatty acid at approximately 45% of total fatty acids. Elevated oleic acid levels are commonly reported in farmed salmon and are mainly attributed to the extensive use of vegetable oils, particularly rapeseed oil, in aquaculture feeds [37]. In addition, PUFAs accounted for 31.30% of total fatty acids in freeze-dried salmon and 33.89% in the oil obtained by supercritical CO_2_ extraction, highlighting the favorable nutritional quality of both lipid fractions. The fatty acid composition obtained in the present study is in close agreement with values reported for North American farmed Atlantic salmon by Langille et al. [39], who reported 49.2% MUFA, 34.2% PUFA, 14.8% n-3 fatty acids and 19.4% n-6 fatty acids, confirming that the analyzed samples exhibit a lipid profile typical of contemporary farmed Atlantic salmon.

The EPA and DHA contents in freeze-dried salmon were 3.09% and 3.34%, respectively, whereas the corresponding values in the oil obtained by supercritical CO_2_ extraction were 2.31% and 3.30%, respectively. Although these values are lower than those typically reported for wild salmon, they are characteristic of contemporary farmed Atlantic salmon. Molversmyr et al. [37] reported EPA and DHA levels of 6.11% and 12.94%, respectively, in wild salmon, compared with 2.19% and 3.94% in farmed salmon, which is in good agreement with the results obtained in the present study. Furthermore, the high proportion of linoleic acid (14.74–15.36%) provides additional evidence of the influence of plant-derived feed ingredients on the lipid profile of the analyzed samples. Megdal et al. [38] identified linoleic acid as one of the most reliable markers for distinguishing farmed from wild salmon, as its proportion is significantly higher in aquaculture-produced fish.

Comparison of freeze-dried salmon and the oil obtained by supercritical CO_2_ extraction revealed a very similar overall fatty acid profile. The proportion of saturated fatty acids remained nearly unchanged (16.19% and 15.72%, respectively), while MUFAs accounted for approximately half of the total fatty acids in both samples. However, the extracted oil contained slightly higher proportions of total PUFAs (33.89%) and omega-3 fatty acids (16.84%) compared with freeze-dried salmon (31.30% and 13.76%, respectively). Supercritical CO_2_ extraction may exhibit a certain degree of selectivity toward different lipid classes and bioactive compounds, potentially resulting in slight changes in fatty acid composition compared to the original raw material [14,15]. The higher proportion of α-linolenic acid (10.98% vs. 7.21%) and total omega-3 fatty acids in the extracted oil is consistent with a relative enrichment of the recovered lipid fraction in unsaturated fatty acids. At the same time, the DHA content remained essentially unchanged, indicating that the extraction process did not result in substantial losses of nutritionally valuable long-chain omega-3 fatty acids.

The omega-6/omega-3 ratio is widely regarded as an important indicator of lipid nutritional quality. Modern diets are typically characterized by a high intake of omega-6 fatty acids, resulting in omega-6/omega-3 ratios frequently exceeding 10:1 and sometimes reaching 20:1, which has been associated with an increased risk of cardiovascular, inflammatory, and metabolic disorders [36]. In the present study, the omega-6/omega-3 ratio was 1.28 in freeze-dried salmon and 1.01 in the oil obtained by supercritical CO_2_ extraction. This ratio was very similar to the omega-6/omega-3 ratio of 1.31 reported by Langille et al. [39] for farmed Atlantic salmon. These values indicate a highly favorable fatty acid composition, characterized by a nearly balanced ratio between omega-6 and omega-3 fatty acids. The lower omega-6/omega-3 ratio observed in the extracted oil is consistent with its higher relative proportion of omega-3 fatty acids. Together with the high proportion of total unsaturated fatty acids, these findings suggest that the extracted oil may be considered a valuable source of essential fatty acids with potential health-promoting properties.

### 3.4. HPLC Determination of Astaxanthin in the SC-CO_2_ Extract

The SC-CO_2_ extract obtained at 300 bar contained 215.43 µg/mL of astaxanthin, a bioactive carotenoid widely studied for its antioxidant and anti-inflammatory properties and its potential role in modulating oxidative stress-related processes [10]. At the molecular level, astaxanthin can quench singlet oxygen and trap free radicals both at the surface and within lipid membranes, thereby limiting lipid peroxidation and membrane oxidative damage [10,40]. Evidence from randomized clinical trials indicates that astaxanthin supplementation may reduce lipid peroxidation and improve selected markers of antioxidant capacity and inflammation [41]. Clinical studies have also suggested potential benefits for skin health, including improved moisture and elasticity and protection against UV-induced damage, supporting its possible use in nutraceutical and cosmeceutical products [42]. Furthermore, preclinical studies have indicated that astaxanthin may exert antiproliferative, pro-apoptotic, and antimetastatic effects in experimental cancer models, particularly in colorectal cancer; however, its anticancer efficacy in humans remains to be established [43,44].

Astaxanthin contents ranging from 5.5 to 8 µg/g have been reported for farmed Atlantic salmon flesh [10]. However, direct numerical comparison with the present result is limited because these literature values refer to whole muscle tissue and are expressed on a mass basis, whereas the present result represents the concentration in a lipid-rich extract expressed on a volume basis. Roy et al. [45] reported an astaxanthin content of 48.20 ± 0.15 µg/g oil in a mixed oil concurrently extracted from tiger shrimp waste and Japanese Spanish mackerel skin using SC-CO_2_. Since astaxanthin is highly lipophilic and its oral bioavailability can be improved when incorporated into lipid-based formulations [46], the lipid-rich nature of the SC-CO_2_ extract obtained in the present study may represent an advantageous matrix for its further utilization. The simultaneous presence of astaxanthin and unsaturated fatty acids supports further evaluation of this extract as a potential bioactive ingredient for nutraceutical and cosmeceutical applications.

### 3.5. ABTS Radical-Scavenging Activity

The SC-CO_2_ extract obtained at 300 bar showed a concentration-dependent increase in ABTS radical-scavenging activity across the tested concentration range (Table 4). Inhibition of the ABTS radical increased from 20.6 ± 2.2% at 40 mg/mL to 53.8 ± 1.1% at 120 mg/mL and linear regression of the concentration–response curve yielded an IC_50_ value of approximately 109 mg/mL. Haq et al. [8] evaluated the ABTS radical-scavenging activity of oils obtained from four Atlantic salmon by-product fractions, including belly part, trimmed muscle, frame bone, and skin, and belly-part oil showed the highest activity (85.18 ± 0.95%). However, direct numerical comparison is limited by differences in raw material composition, sample concentration, and assay conditions. The observed ABTS radical-scavenging activity may be partly associated with the astaxanthin detected in the SC-CO_2_ extract, although other lipid-soluble constituents may also have contributed to the overall activity [14,47]. Moreover, no standardized reference ranges are currently available for classifying the ABTS radical-scavenging activity of fish oils as high or moderate, since reported values strongly depend on the experimental protocol and the manner in which antioxidant activity is expressed. Although the assay was performed at room temperature in the dark to minimize oxidative changes, potential auto-oxidation of unsaturated lipids during the prolonged incubation cannot be completely excluded and should be acknowledged as a methodological limitation when interpreting the ABTS radical-scavenging results.

### 3.6. Yield of Protein Extraction

In recent times, the pH shift process has been widely used in recovering functional protein isolates from aquatic products and their processing wastes [19]. The pH-shift technology emerges as a promising solution for handling complex and even unsorted seafood side streams [48]. Yields of proteins extracted from salmon by-products using the pH shift method (SPI-pH) and ultrasound- assisted pH shift method (SPI-USpH) are presented in Table 5. Using the pH-shift method, a total protein yield of 37% was obtained from salmon residues at a solubilization pH of 11, indicating that alkaline conditions promoted effective protein extraction and recovery from the processing residues.

Similar results of total protein yields have been reported for the pH-shift method of by-products from different fish species. Reported protein recoveries vary considerably among fish by-products, with values of 68.52% for Pangas (*Pangasius pangasius*) frames [49], approximately 32% for Rohu processing waste [50], 31% for frozen croaker [51], up to 60.4% for herring (*Clupea harengus*) by-products [52], and up to 68% for tilapia (*Oreochromis niloticus*) [53]. Differences in protein yield can be influenced by three major factors: the solubility of the proteins at extreme acid or alkaline conditions, the size of the sediments formed during the separation process, and the solubility of the proteins at the pH selected for precipitation [18].

Results showed that ultrasound-assisted pH-shift processing significantly increased total protein yield (47%) compared to the pH-shift method. The increase in protein yield can be attributed to ultrasound treatment, which promoted the release and solubilization of the proteins from the insoluble salmon residues, thereby reducing protein entrapment in sediment [20]. A synergistic combination of ultrasonication and pH-shift processing has been reported to significantly enhance the extraction yield of proteins isolated from rainbow trout by-products while also improving the functional properties of this protein isolate [20].

### 3.7. Chemical Composition

The chemical composition of proteins obtained from salmon residues by the pH shift method and the combination of ultrasound and pH shift method is presented in Table 6.

Results demonstrated that both proteins contained more than 90% protein, indicating a high degree of purity and classifying these proteins as protein isolates. Similarly, proteins recovered from Cape hake by-products using the pH shift method contained ~90% protein on a dry-weight basis, confirming the efficiency of the extraction process and high purity [54]. Moisture content of SPI-pH and SPI-USpH were 6.7 and 5.4%, respectively, which are lower than those reported for dried fish protein isolates produced from salmon, cod and herring by-products [17]. Protein isolate obtained using the ultrasound-assisted pH shift method showed a higher ash content than the isolate produced by the pH shift method; however, both values were consistent with those reported in the literature [55,56].

### 3.8. Amino Acid Composition

It is well known that each protein has a unique amino acid composition, which determines its properties as well as its nutritional quality. Low-quality protein does not contain all the essential amino acids needed for protein synthesis, whereas high-quality protein contains most or all essential amino acids required for human body function. To further investigate the quality of proteins in the obtained samples, the amino acid composition was determined. The amino acid compositions of proteins produced by the pH shift method and the combination of ultrasound and the pH shift method are shown in Table 7.

The sum of essential amino acids showed similar values in both protein samples, accounting for approximately 41% of the amino acid composition in both SPI-pH and SPI-USpH. The relatively high proportion of essential amino acids suggests favorable nutritional potential, although a detailed comparison with the FAO reference amino acid pattern [57] is required to fully assess protein quality. The results revealed no statistically significant differences (*p* > 0.05) in the individual amino acid contents between the two protein isolates, suggesting that ultrasound-assisted pH-shift processing did not substantially affect their amino acid profiles. The results showed that glutamic acid (~13 g/100 g DM of protein isolate) and aspartic acid (~11 g/100 g DM of protein isolate) were the predominant amino acids in all isolates, which is consistent with literature reports identifying them as the major amino acids in fish proteins [58]. Lysine and leucine were the next most abundant amino acids, which is typical for proteins from aquatic species [59]. Cysteine was not detected in either protein isolate. The absence of detectable cysteine is consistent with previous reports indicating its low abundance in fish proteins [60,61]. Recent findings proved that proteins derived from fish have a well-balanced amino acid profile what makes them an important source of quality proteins in human nutrition.

### 3.9. FTIR Analysis of Protein Isolates

**Figure 1:**
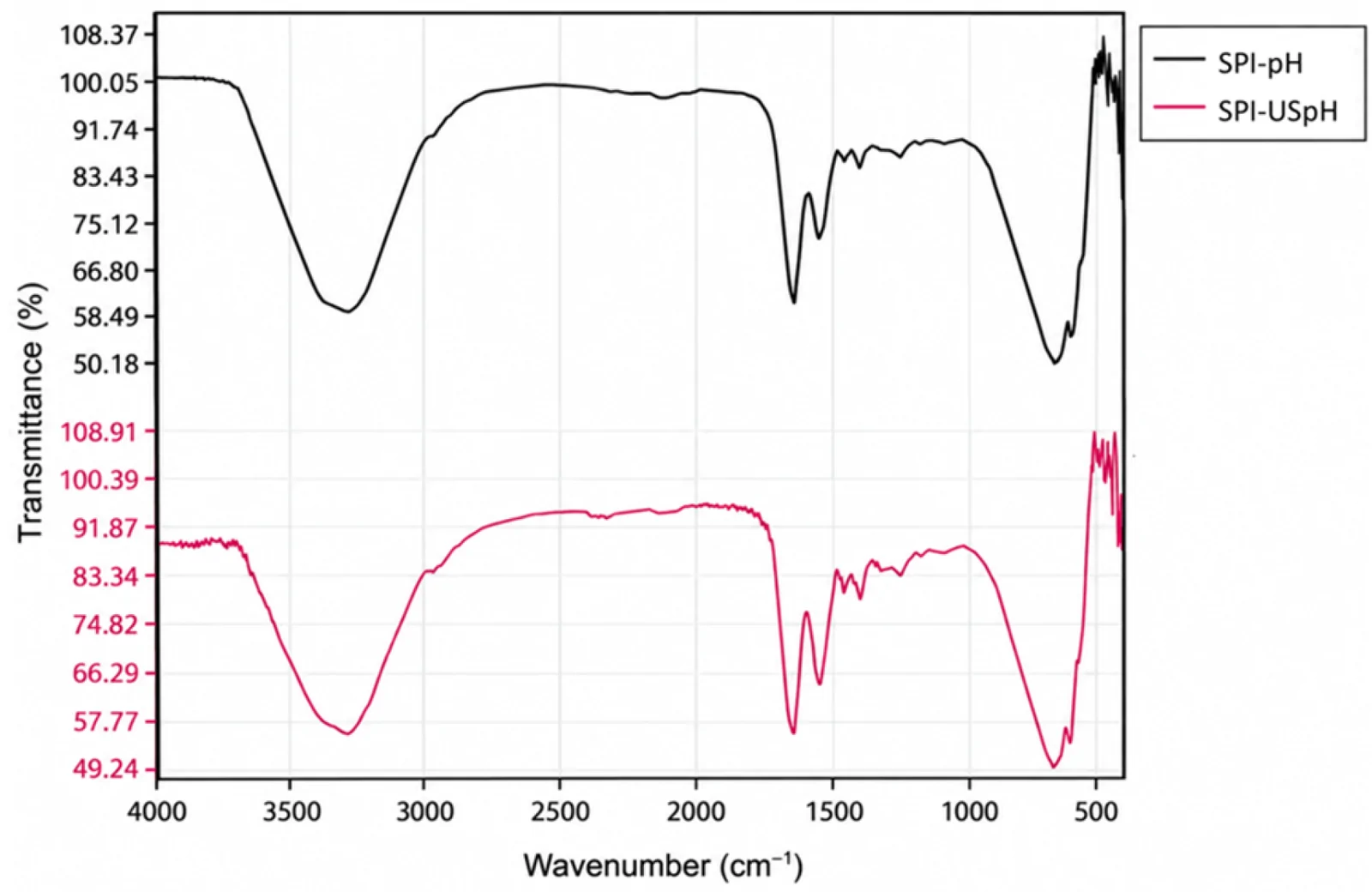
FTIR spectra of protein isolates obtained by pH-shift processing (SPI-pH; spectrum 1) and ultrasound-assisted pH-shift processing (SPI-USpH; spectrum 2).

Figure 1 shows the FTIR spectra of the protein isolates obtained by pH-shift processing (SPI-pH) and ultrasound-assisted pH-shift processing (SPI-USpH). Two peaks between 3600 and 3000 cm^−1^ are attributed to the Amide A (N–H stretching vibration), overlapping with hydrogen-bonded O–H stretching. Weak bands between 2930 and 2850 cm^−1^ correspond to CH2 and CH cm^−1^ stretching vibrations. A strong band at 1654 cm^−1^ is assigned to the amide I band (C=O stretching vibration), suggesting predominance of α-helix structure. The band at 1540 cm^−1^ is attributed to the Amide II (N–H bending coupled with C–N stretching). The absorption band at 1397 cm^−1^ is attributed to the symmetric stretching vibration of carboxylate (COO^−^) groups, mainly arising from the side chains of amino acids. The band observed at 1243 cm^−1^ corresponds to the Amide III region, which involves coupled C–N stretching and N–H bending vibrations and provides additional information about the protein’s secondary structure. The band around 669 cm^−1^ can be assigned to out-of-plane bending vibrations, potentially associated with aromatic amino acid residues or skeletal vibrational modes of the protein backbone. A band around 600 cm^−1^ is attributed to the out-of-plane bending of aromatic C–H bonds.

### 3.10. Functional Properties

In addition to their biological activity and important nutritional role, proteins also make a significant contribution to the technological and sensory characteristics of various food and pharmaceutical products. Functional properties are directly related to the physical, chemical, and conformational characteristics of proteins, which primarily include molecular size and shape, amino acid composition and sequence, as well as the presence and distribution of electrical charges. The functional properties of the two prepared protein isolates were determined in order to assess the possible influence of the extraction method, which could be important for their further implementation.

#### 3.10.1. Solubility

Protein solubility is an important functional characteristic that determines its suitability for a wide range of applications. The obtained results for the solubility of the proteins produced from salmon by-products are presented in Table 8. The results showed that there were significant differences in the solubility of the isolated fish proteins. At pH 9, the highest solubility (approximately 95%) was measured for the protein obtained by combination of ultrasound and pH shift method, whereas the lower solubility (approximately 80%) was recorded for the protein obtained by pH shift method only.

Solubility of proteins in water depends on several factors, including surface characteristics of their amino acids, molecular weight, and conformational situation [62]. Compared with previous findings, the proteins analyzed in this study exhibited markedly higher solubility than cod and herring proteins, which showed solubility values of 53% and 29%, respectively, at pH 10 [16]. Prepared salmon proteins exhibited higher solubility compared to soy protein isolate, indicating their potential suitability for applications comparable to those of soy proteins in food systems [16].

#### 3.10.2. Water/Oil Holding Capacity

Water-holding capacity (WHC) represents the mass of water that can be retained per gram of protein. The hydration properties of proteins determine their applications in different systems. WHC is primarily determined by the inherent properties of the protein molecule, but is also influenced by factors related to the medium in which the protein is dispersed [63]. The results of this functional property revealed that the water-holding capacity of protein isolate obtained by ultrasound-assisted extraction was higher than that of isolate produced using the pH-shift method (Table 8). The higher WHC of the ultrasound-assisted isolate may be attributed to ultrasound-induced partial unfolding of protein molecules, which exposes additional groups and enhances protein–water interactions. Furthermore, a possible reduction in particle size and an increase in surface area can facilitate water retention within the protein matrix [64].

Oil-holding capacity (OHC) is another functional property of proteins that is of fundamental importance for their implementation in various products. Results showed that both protein isolates exhibited good oil-holding capacity (Table 8). The OHC value of the SPI-pH isolate was higher but comparable to those previously reported in the literature, regardless of whether the protein was from finfish and shellfish wastes [65] or hake and mackerel [66]. As both protein isolates exhibited a comparable amino acid composition, the differences observed in WHC and OHC are more likely associated with structural and conformational changes induced during the extraction process rather than differences in protein composition.

#### 3.10.3. Antiradical Scavenging Activity

**Figure 2:**
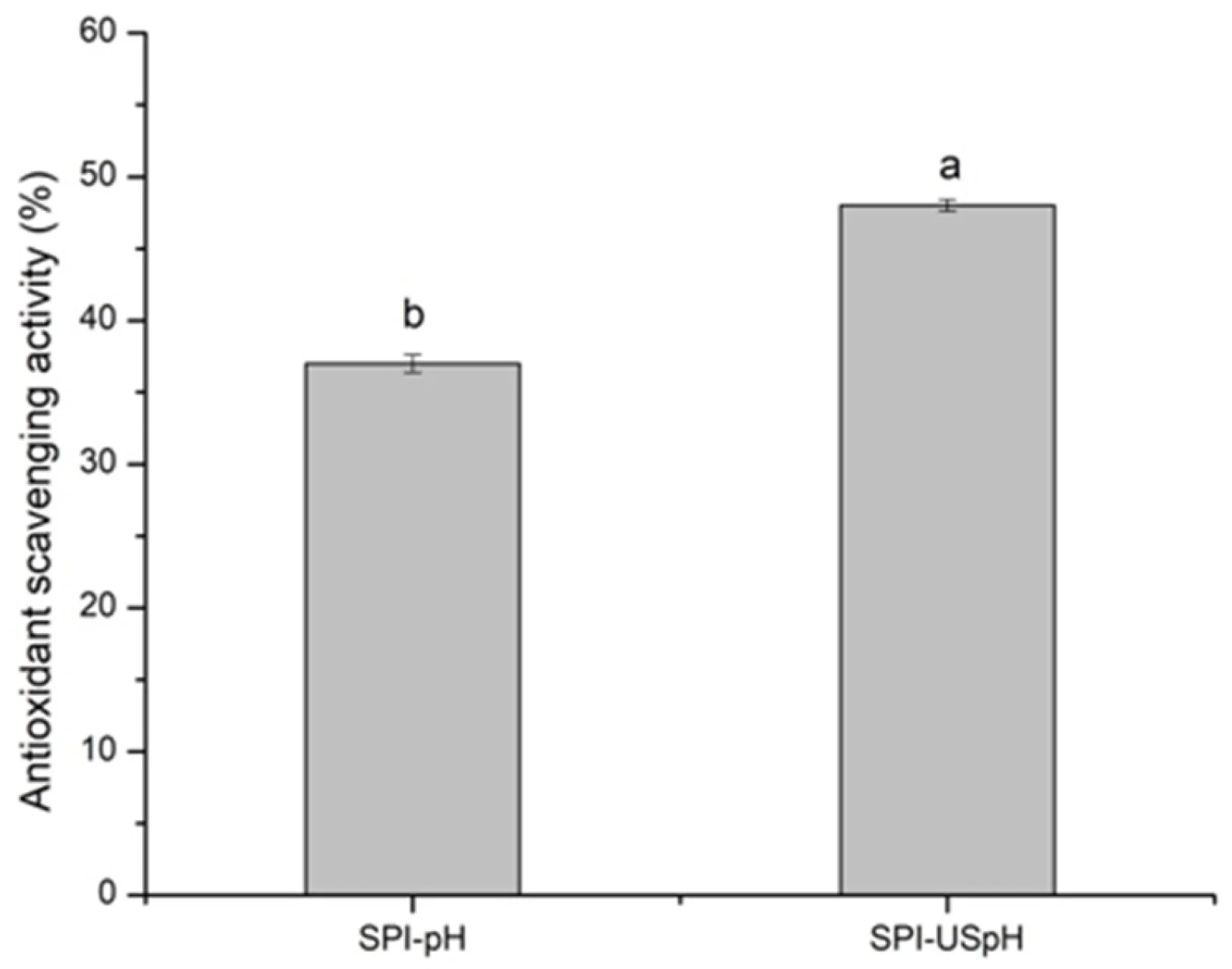
Antiradical scavenging activity of proteins obtained by the pH shift method and the combination of ultrasound and the pH shift method. Different letters indicate statistically significant differences according to Tukey’s test (*p* < 0.05).

In addition to their functional properties, proteins also possess considerable potential as antioxidants due to their capacity to scavenge free radicals through various mechanisms [66]. Protein isolates obtained after the pH method, with and without ultrasound pretreatment, were analyzed using the ABTS assay to determine their in vitro antioxidant activity, and the results are presented in Figure 2.

Results showed that antioxidant activity of the protein isolate obtained by the ultrasound-assisted pH shift method (48%) was higher compared with that obtained from the pH shift method only (37%). The increase observed following ultrasound treatment can be explained by partial unfolding of the protein structure, which enhances the accessibility of the solvent to amino acid side chains capable of scavenging free radicals [67]. Moreover, it is well established that increased protein solubility is associated with enhanced antioxidant activity, as it promotes more efficient interactions between antioxidants and reactive radical species [68]. Accordingly, the results obtained for SPI-USpH are consistent with this relationship. Similar results were reported by Nasyiruddin et al. [69], who demonstrated that protein extracted from silver carp using the ultrasound-assisted pH shift method at pH 11 exhibited higher antioxidant activity compared to protein obtained using the pH shift method at the same pH level.

## 4. Conclusions

The sequential combination of SC-CO_2_ extraction and pH-shift processing enabled the recovery of lipid- and protein-rich fractions from the same Atlantic salmon skin-on trimmings. Among the tested SC-CO_2_ conditions, 300 bar provided the highest extraction yield (28.08%), and the resulting lipid-rich extract contained unsaturated fatty acids, including EPA and DHA, as well as astaxanthin and measurable radical-scavenging activity. Subsequent ultrasound-assisted pH-shift processing increased protein recovery from 36.70 to 47.91 g/100 g while maintaining a protein content above 91 g/100 g and a balanced amino acid composition, and improving solubility, water-holding capacity, and antioxidant activity. These findings support the feasibility of the proposed sequential valorization strategy. Future studies should employ a formal experimental design to optimize SC-CO_2_ extraction and protein-recovery conditions and include a comprehensive process mass balance accounting for all recovered and residual streams. This would enable a more complete assessment of overall process efficiency and resource utilization, together with further evaluation of process scale-up, product stability, and potential applications.

## Figures and Tables

**Table 1:** Extraction yield obtained during supercritical extraction (%).

Time (min)	100 Bar	200 Bar	300 Bar
30	0.53 ± 0.04 ^p^	0.61 ± 0.01 ^p^	3.14 ± 0.10 ^k–n^
60	0.82 ± 0.03 ^op^	1.22 ± 0.04 ^m–p^	6.20 ± 0.13 ^ij^
90	1.11 ± 0.09 ^nop^	1.84 ± 0.17 ^L–p^	8.51 ± 0.25 ^gh^
120	1.17 ± 0.04 ^nop^	2.41 ± 0.11 ^L–p^	11.16 ± 0.40 ^f^
180	1.67 ± 0.10 ^L–p^	3.7 ± 0.15 ^kL^	15.92 ± 0.38 ^e^
240	1.85 ± 0.14 ^L–p^	4.78 ± 0.19 ^jk^	18.81 ± 1.65 ^d^
300	2.34 ± 0.09 ^L–p^	5.97 ± 0.20 ^ij^	21.16 ± 0.63 ^c^
360	2.59 ± 0.11 ^k–p^	7.3 ± 0.63 ^hi^	24.38 ± 2.32 ^b^
420	2.91 ± 0.06 ^k–o^	7.55 ± 0.59 ^ghi^	25.99 ± 1.09 ^ab^
480	3.12 ± 0.22 ^k–n^	8.55 ± 0.27 ^gh^	27.62 ± 2.55 ^a^
540	3.47 ± 0.10 ^klm^	9.65 ± 0.65 ^fg^	28.08 ± 2.15 ^a^

Values are expressed as mean ± standard deviation (SD) of three independent extraction runs (*n* = 3). Different superscript letters indicate statistically significant differences among experimental conditions according to Tukey’s post hoc test (*p* < 0.05).

**Table 2:** Composition of freeze-dried salmon skin-on trimmings and the SC-CO_2_ extract obtained at 300 bar.

Component (g/100 g Sample)	Raw Material	SC-CO_2_ Extract
**Moisture**	1.07 ± 0.18 ^a^	0.76 ± 0.05 ^b^
**Protein**	54.42 ± 0.12 ^a^	0.46 ± 0.03 ^b^
**Lipids**	40.14 ± 0.07 ^b^	98.55 ± 0.12 ^a^
**Ash**	3.95 ± 0.09	ND
**Carbohydrates**	0.42 ± 0.03 ^a^	0.24 ± 0.08 ^b^

Values are expressed as mean ± standard deviation (SD) of three replicates (*n* = 3) on an as-is basis. Different superscript letters within the same row indicate statistically significant differences between samples (*p* < 0.05). ND—not detected (below the method detection limit).

**Table 3:** Fatty acid composition of the freeze-dried raw material and the SC-CO_2_ extract obtained at 300 bar.

Fatty Acid Composition (% of Total Fatty Acids)			
Fatty Acid		Raw Material	SC-CO_2_ Extract
**C12:0**	Lauric acid	ND	0.03 ± 0.00
**C14:0**	Myristic acid	2.21 ± 0.02 ^a^	1.75 ± 0.03 ^b^
**C14:1**	Myristoleic acid	ND	0.01 ± 0.00
**C15:0**	Pentadecanoic acid	0.17 ± 0.00 ^a^	0.15 ± 0.01 ^b^
**C16:0**	Palmitic acid	10.37 ± 0.02 ^a^	9.01 ± 0.04 ^b^
**C16:1**	Palmitoleic acid	2.76 ± 0.02 ^a^	2.11 ± 0.06 ^b^
**C17:0**	Heptadecanoic acid (Margaric acid)	0.16 ± 0.01 ^b^	0.32 ± 0.01 ^a^
**C17:1**	Heptadecenoic acid	0.13 ± 0.00 ^a^	0.12 ± 0.01 ^a^
**C18:0**	Stearic acid	2.76 ± 0.02 ^a^	2.98 ± 0.05 ^a^
**C18:1n9c**	Oleic acid	45.43 ± 0.08 ^a^	45.41 ± 0.04 ^a^
**C18:2n6c**	Linoleic acid	15.36 ± 0.02 ^a^	14.74 ± 0.04 ^a^
**C20:0**	Arachidic acid	0.34 ± 0.02 ^b^	0.46 ± 0.03 ^a^
**C18:3n6**	γ-Linolenic acid (GLA)	0.10 ± 0.01 ^b^	0.23 ± 0.01 ^a^
**C18:3n3**	α-Linolenic acid (ALA)	7.21 ± 0.03^b^	10.98 ± 0.06 ^a^
**C20:1n9**	Gondoic acid (Eicosenoic acid)	1.38 ± 0.04	ND
**C20:2n6**	Eicosadienoic acid	1.52 ± 0.02 ^a^	1.34 ± 0.03 ^a^
**C22:0**	Behenic acid	0.14 ± 0.00 ^a^	0.14 ± 0.01 ^a^
**C20:3n6**	Dihomo-γ-linolenic acid (DGLA)	0.17 ± 0.01 ^b^	0.25 ± 0.01 ^a^
**C22:1n9**	Erucic acid	0.93 ± 0.02 ^b^	1.39 ± 0.04 ^a^
**C20:4n6**	Arachidonic acid (ARA)	0.39 ± 0.02 ^b^	0.49 ± 0.03 ^a^
**C23:0**	Tricosanoic acid	ND	0.85 ± 0.03
**C20:5n3**	Eicosapentaenoic acid (EPA)	3.09 ± 0.05 ^a^	2.31 ± 0.02 ^b^
**C24:1**	Nervonic acid	0.26 ± 0.00 ^b^	0.40 ± 0.01 ^a^
**C22:5n3**	Docosapentaenoic acid (DPA, n-3)	0.14 ± 0.01 ^b^	0.25 ± 0.01 ^a^
**C22:6n3**	Docosahexaenoic acid (DHA)	3.34 ± 0.04 ^a^	3.30 ± 0.06 ^a^
	Unidentified fatty acid	1.61 ± 0.09 ^a^	0.97 ± 0.09 ^b^
**Fatty acid groups and nutritional indices:**			
**ΣSFA**		16.19 ± 0.06 ^a^	15.72 ± 0.08 ^a^
**ΣMUFA**		50.91 ± 0.21 ^a^	49.41 ± 0.07 ^a^
**ΣPUFA**		31.30 ± 0.04 ^b^	33.89 ± 0.65 ^a^
**Σn-3 PUFA**		13.76 ± 0.03 ^a^	16.84 ± 0.03 ^a^
**Σn-6 PUFA**		17.54 ± 0.02 ^a^	17.06 ± 0.13 ^a^
**omega-6/omega-3 ratio**		1.28 ± 0.01 ^a^	1.01 ± 0.01 ^b^

Values are expressed as mean ± standard deviation (*n* = 3). ND, not detected; SFA, saturated fatty acid; MUFA, monounsaturated fatty acid; PUFA, polyunsaturated fatty acid. Different letters represent statistically significant differences determined by Tukey’s test at the *p* < 0.05 level.

**Table 4:** ABTS radical-scavenging activity of the SC-CO_2_ extract obtained at 300 bar.

Concentration (mg/mL)	ABTS Inhibition (%)
**40**	20.6 ± 2.2
**60**	30.1 ± 2.7
**80**	37.3 ± 1.4
**100**	46.6 ± 0.1
**120**	53.8 ± 1.1

Values are expressed as mean ± standard deviation (*n* = 3).

**Table 5:** Yield of proteins extracted from salmon residues using pH shift method and ultrasound- assisted pH shift method.

	Yield (g/100 g)
SPI-pH	36.70 ± 0.11 ^b^
SPI-USpH	47.91 ± 0.47 ^a^

Different letters represent statistically significant differences determined by Tukey’s test at the *p* < 0.05 level.

**Table 6:** Chemical composition of proteins extracted from salmon residues using the pH shift method and the ultrasound-assisted pH shift method.

	SPI-pH	SPI-USpH
Protein	92.55 ± 0.40 ^a^	91.35 ± 0.24 ^a^
Moisture	6.71 ± 0.11 ^a^	5.38 ± 0.20 ^b^
Ash	1.71 ± 0.16 ^b^	4.58 ± 0.01 ^a^

Different letters represent statistically significant differences determined by Tukey’s test at *p* < 0.05.

**Table 7:** Amino acid composition of proteins obtained by the pH shift method and the combination of ultrasound and the pH shift method.

Content g/100 g	SPI-pH	SPI-USpH
Asp	11.49 ± 0.05 ^a^	11.32 ± 0.04 ^a^
Thr	4.44 ± 0.02 ^a^	4.53 ± 0.00 ^a^
Ser	4.61 ±0.00 ^a^	4.51 ± 0.06 ^a^
Glu	13.77 ± 0.04 ^a^	13.79 ± 0.02 ^a^
Pro	2.23 ± 0.00 ^a^	2.16 ± 0.03 ^a^
Gly	4.16 ± 0.01 ^a^	4.21 ± 0.01 ^a^
Ala	5.86 ± 0.00 ^a^	5.75 ± 0.04 ^a^
Cys	ND	ND
Val	5.67 ± 0.05 ^a^	5.71 ± 0.03 ^a^
Met	3.73 ± 0.00 ^a^	3.85 ± 0.02 ^a^
Ile	4.45 ± 0.01 ^a^	4.48 ± 0.00 ^a^
Leu	6.68 ± 0.06 ^a^	6.71 ± 0.01 ^a^
Tyr	3.17 ± 0.00 ^a^	3.13 ± 0.02 ^a^
Phe	3.99 ± 0.05 ^a^	3.96 ± 0.02 ^a^
His	2.51 ± 0.02 ^a^	2.54 ± 0.00 ^a^
Lys	9.57 ± 0.03 ^a^	9.43 ± 0.03 ^a^
Arg	5.41 ± 0.02 ^a^	5.21 ± 0.01 ^a^

Values are expressed as mean ± standard deviation (*n* = 3). ND, not detected. Different letters represent statistically significant differences determined by Tukey’s test at the *p* < 0.05 level.

**Table 8:** Solubility, WHC and OHC of proteins obtained by pH shift method and combination of ultrasound and pH shift method.

	SPI-pH	SPI-USpH
Solubility (%)	79 ± 0.04 ^b^	95 ± 0.08 ^a^
WHC	4.00 ± 0.05 ^b^	4.26 ± 0.05 ^a^
OHC	3.27 ± 0.09 ^a^	2.44 ± 0.24 ^b^

Different letters represent statistically significant differences determined by Tukey’s test at the *p* < 0.05 level.

## Data Availability

The original contributions presented in this study are included in the article. Further inquiries can be directed to the corresponding author.
